# The impact of utilizing oyster shell soil conditioner on the growth of tomato plants and the composition of inter-root soil bacterial communities in an acidic soil environment

**DOI:** 10.3389/fmicb.2023.1276656

**Published:** 2024-01-16

**Authors:** Yi Zheng, Chaofan Yu, Yujun Xiao, Tinge Ye, Songgang Wang

**Affiliations:** ^1^College of Life Sciences, Fujian Normal University, Fuzhou, Fujian, China; ^2^National Joint Engineering Research Center of Industrial Microbiology and Fermentation Technology, College of Life Sciences, Fujian Normal University, Fuzhou, Fujian, China

**Keywords:** oyster shell soil conditioner, acidic red loam soil, tomato, soil physicochemical, bacterial flora structure

## Abstract

**Introduction:**

The objective of this study is to examine the impact of various oyster shell soil conditioners, which are primarily composed of oyster shells, on the growth of tomatoes in acidic soil. Moreover, the aim of this investigation is to analyze the variety and structure of soil bacterial populations in close proximity to tomato roots while also contributing to the understanding of the physical, chemical, and biological mechanisms of oyster shell soil conditioners.

**Methods:**

Tomato plants were grown in acidic red soil in three groups: a control group and a treatment group that used two types of oyster shell soil conditioners, OS (oyster shell powder) and OSF (oyster shell powder with organic microbial fertilizer). A range of soil physicochemical properties were measured to study differences in inter-soil physicochemical parameters and the growth of tomato plantings. In addition, this study utilized the CTAB (Cetyltrimethylammonium Bromide) technique to extract DNA from the soil in order to investigate the effects of oyster shell soil conditioner on the composition and diversity of bacterial populations. Utilizing high-throughput sequencing technologies and diversity index analysis, the composition and diversity of bacterial populations in the soil adjacent to plant roots were then evaluated. Ultimately, correlation analysis was used in this study to explore the relationship between environmental factors and the relative abundance of soil bacteria in the inter-root zone of tomato plants.

**Results:**

The findings indicated that the oyster shell soil conditioners were capable of modifying the physicochemical characteristics of the soil. This was evidenced by significant increases in soil total nitrogen (16.2 and 59.9%), soil total carbon (25.8 and 27.7%), pH (56.9 and 55.8%), and electrical conductivity (377.5 and 311.7%) in the OS and OSF groups, respectively, compared to the control group (*p* < 0.05). Additionally, data pertaining to tomato seed germination and seedling growth biomass demonstrated that both oyster shell soil conditioners facilitated the germination of tomato seeds and the growth of seedlings in an acidic red clay soil (p < 0.05). On the other hand, the application of two oyster shell soil conditioners resulted in a modest reduction in the diversity of inter-root soil bacteria in tomato plants. Specifically, the group treated with OSF exhibited the most substantial fall in the diversity index, which was 13.6% lower compared to the control group. The investigation carried out on the soil between tomato plant roots yielded findings about the identification of the ten most abundant phyla. These phyla together represented 91.00-97.64% of the overall abundance. In the inter-root soil of tomatoes, a study identified four major phyla, namely *Proteobacteria, Bacteroidetes, Acidobacteria*, and *Actinobacteria*, which collectively accounted for up to 85% of the total abundance. At the general level, the relative abundance of *Massilia* increased by 2.18 and 7.93%, *Brevundimonas* by 5.43 and 3.01%, and *Lysobacter* by 3.12 and 7.49% in the OS and OSF groups, respectively, compared to the control group. However, the pathogenic bacteria *unidentified_Burkholderiaceae* decreased by 5.76 and 5.05%, respectively. The correlation analysis yielded conclusive evidence indicating that, which involved the use of CCA (Canonical Correlation Analysis) graphs and Spearman correlation coefficients, pH exhibited a positive correlation (*p* < 0.05) with *Shewanella* and a negative correlation (*p* < 0.05) with *Bradyrhizobium*. The relative abundance of *Lysobacter* and *Massilia* exhibited a positive correlation with the levels of total soil nitrogen.

**Discussion:**

The utilization of oyster shell soil conditioner on acidic red soil resulted in several positive effects. Firstly, it raised the pH level of the inter-root soil of tomato plants, which is typically acidic. This pH adjustment facilitated the germination of tomato seeds and promoted the growth of seedlings. In addition, the application of oyster shell soil conditioner resulted in changes in the structure of the bacterial community in the inter-root soil, leading to an increase in the relative abundance of *Proteobacteria* and *Bacteroidetes* and a decrease in the relative abundance of *Acidobacteria*. Furthermore, this treatment fostered the proliferation of genera of beneficial bacteria like *Massilia*, *Brevundimonas*, and *Lysobacter*, ultimately enhancing the fertility of the red soil.

## 1 Introduction

Soils exhibiting a pH level lower than 6.5 are categorized as acidic (Huang, 2000). In typical scenarios, the process of soil acidification occurs gradually over a span of around 100 years (Krug and Frink, 1983). In recent years, there has been a significant increase in the expansion of facility-based agriculture (Shen, 1999). The process of reducing soil acidity has been expedited by the expansion, in conjunction with factors such as limited precipitation and specific environmental conditions prevalent in protected areas. These circumstances include high indoor air temperatures, high humidity, inadequate aeration, and heavy irrigation (Li and Zhang, 2001). Consequently, this has led to the emergence of various soil-related issues (Jiang et al., 2005; Wang et al., 2005; Deng and Zhang, 2006). Each plant species has evolved to thrive within a specific soil pH range, which is considered best for their growth throughout a significant duration of evolutionary history (Gilroy et al., 1989). Soil acidification causes soil surface consolidation, which prevents the soil root system from extending, resulting in a number of phenomena such as a decrease in the number of roots, slow growth, and reduced plant growth (Zeng, 2018), as well as a large loss of effective soil nutrients, resulting in Al^3+^ enrichment and toxic effects, severely affecting crop yield in the region (Xie, 2019). According to research, soil pH influences the relative number and variety of microorganisms (Zhalnina et al., 2015). The inter-root soil is home to a plethora of microorganisms, including bacteria, fungus, actinomycetes, and protozoa, with bacteria playing an important role in soil fertility, plant growth and development, and nutrient absorption by plant roots (Dotaniya and Meena, 2015; Zhang W. et al., 2019). Inter-rhizosphere bacteria can affect the efficacy of nitrogen, phosphorus, and trace metals, including iron, manganese, zinc, and copper, for plants and soil microbial communities, in addition to the geochemical cycling of these elements. By enhancing the nutritional content of plants, stimulating the production of growth hormones, or serving as a biological agent for disease control, it effectively enhances agricultural yields (Dotaniya and Meena, 2015).

Oysters serve as a crucial marine resource that is utilized by human populations (Southgate and Lucas, 2008; Zhou et al., 2014). Oysters are widely recognized as a highly valuable marine resource in terms of their suitability for human consumption. China is first in terms of oyster production among countries and areas engaged in oyster cultivation, with Korea, Japan, the United States, and France following suit (Zhang, 2014). Throughout an extended period of evolutionary time, each plant species has acquired a distinct and ideal soil pH for its growth. The oyster farming areas in China are all located along the coast, and the main production areas for Chinese oysters are in Fujian, Guangdong, and Shandong, with Fujian having the longest history of oyster farming and currently being the largest oyster farming area in China (Li et al., 2017; Lin et al., 2019). Currently, oysters are predominantly used for their edible component, while the shell, which accounts for 60% of their volume, is underutilized (Zhao et al., 2015). Recent research has demonstrated that oyster shells play a significant role in the improvement of acidic soils. Li (2019) observed that a homemade oyster shell soil conditioner could enhance the acidic soil of Guanxi honeydew, change the compacted soil into a crop-growing state, and raise soil nutrients from class IV to class III. Lee et al. (2008) applied oyster shell powder to the soil and planted cabbage seedlings to study the impact of oyster shell powder on enhancing the physicochemical qualities and crop yield in chalky loam (SiL, pH 6.2) and sandy loam (SL, pH 5.8) soils. They discovered that oyster shell significantly increased the soil pH to 6.9 and 7.4. Strictly established (Yan, 2019) by applying base fertilizer and oyster shell soil conditioner at 2250 and 1500 kg/hm^2^ to peanuts grown in yellow clay fields, flower production increased by 16.8 and 10.1%, respectively. The soil pH also increased by 0.8 and 0.5, and the soil’s organic, effective phosphorus, alkaline nitrogen, exchangeable calcium, and rapid-efficiency magnesium content were significantly increased. The oyster shell can boost soil organic, rapid phosphorus, and exchange ion concentrations, as well as significantly raise soil microbial carbon and nitrogen content, stimulate soil enzyme activity, and increase crop production (Lee et al., 2008).

In prior studies, our research team has successfully created two soil conditioner products derived from oyster shells. The first product, referred to as OS (Oyster shell powder), is produced by subjecting oyster shell powder to a thermal modification process at a temperature of 700°C and then sieving it through a 300-mesh screen. The second product, known as OSF (Oyster shell powder with organic microbial fertilizer), is a composite material consisting of OS combined with an organic microbial fertilizer.

This study aimed to examine the impact of oyster shell conditioner on tomato growth and the bacterial community in acidic inter-root soil. High-throughput sequencing was employed to analyze the red loam soil samples collected from Wenwu Xuefeng Farm in Minhou, Fujian Province, China. This study holds considerable importance in the comprehensive assessment of the impact of oyster shell conditioner on the bacterial community in acidic inter-root soil. This study provides theoretical support for the application of oyster shell conditioner as a means to improve acidic soil conditions, based on insights from microbiological viewpoints.

## 2 Research materials and methods

### 2.1 Overview of the study area

The soil used in this study was collected from Wenwu Xuefeng Farm, located in Minhou County, Fuzhou City, Fujian Province. The geographical coordinates of the farm are 118°51′ 119°25′ E and 25°47′ 26°37′ N. The climate in this region is characterized by a subtropical monsoon climate, with an annual precipitation of 1,673.9 mm and an average annual temperature ranging from 18 to 26°C. The topography exhibits a threshold characteristic, the climatic conditions are of a moderate nature, and the soil composition is notably characterized by a high level of acidity. The soil’s pH level is approximately 4.8. The experimental site is situated within the Qishan campus of Fujian Normal University in Fuzhou City, Fujian Province. Geographically, it is positioned at coordinates 26°03′ N and 119°20′ E. The region has a subtropical monsoon climate characterized by an average annual temperature of 19.6°C and an annual precipitation of 1674 mm.

### 2.2 Applicators and application methods

The experiment utilized 200 g of red loam soil obtained from Wenwu Xuefeng Farm as the foundational soil. The control group CK did not receive any treatment, while the experimental groups I and II were treated with oyster shell soil conditioner products OS and OSF, respectively. The laboratory independently prepared OS products by subjecting oyster shells to thermal modification at a temperature of 700°C. The resulting shells were subsequently crushed to obtain pure oyster shell powder, which passed through a 300-mesh sieve. The primary constituents of this powder were found to be CaCO3 (90−95%) and CaO (3−5%). OSF products are based on OS products, added to Fujian Hengxiang Fisheries Company’s commercialized organic microbial fertilizer products at 50:1 (w/w). The main components of organic microbial fertilizer are the mass fraction of total nutrients (N+P2O5+K2O) ≥ 4.0% and the number of effective live cells (cfu) ≥ 1 × 108/mL. Mix them thoroughly and place them in a 5 cm tall by 10 cm wide receptacle. Add deionized water to 60−70% of the soil’s normal water content and compact the soil. Then, apply oyster shell soil conditioner at a rate of 1.5 percent of the soil’s weight. Tomato seeds were submerged in water and grown for 24 h. The tomato seeds were immersed in water and cultivated for a duration of 24 h. The seeds were subsequently dispersed evenly across the surface of the soil. Each treatment consisted of 30 One Pine Red Israel Hard Fruited Tomato F1 (Green Bar Seedling Co., Guangzhou) seeds, with three replications being established. After sowing, a layer of dry soil measuring 0.5 cm was used to cover the seeds. Subsequently, a small amount of water was sprayed onto the soil using a watering can. Subsequently, the pots were placed within an incubator set at a temperature of 25°C, and a daily rehydration process was carried out. The experimental groups’ locations were randomly changed at various intervals during the course of the trial.

### 2.3 Measurement of tomato growth indicators

1. Determination of tomato germination potential, germination rate, and germination index The tomato germination cycle endured 8 days, and the number of seeds that germinated in each experimental group was measured daily, with day 5 being the day of germination potential measurement (Xu, 2019) and day 8 being the day of germination rate measurement; the germination potential, germination rate, and germination index were then calculated. Germination potential (%) = (Number of generates on a specified day/total number of seeds) × 100; Germination rate = (total number of germinations in the germination cycle/total number of seeds) × 100;
  $$\text{germination}\text{index}\left(\%\right)=\sum \frac{G_{t}}{D_{t}}$$
  In the equation, Gt is the number of germination days in the final period of the germination test, and Dt is the number of germination days.
2. determining the height, root length, plant fresh weight, root fresh weight, plant dry weight, and root dry weight of tomato vegetation: Five plants of uniform growth were taken from each treatment on the 30th day of incubation, and their plant height and root length were measured with a ruler; the fresh weight of the plants and the fresh weight of the roots were weighed on a one-in-ten-thousand balance; the plants were separated from the roots with scissors, sterilized at 105°C for 5 min, and then dried at 60°C to a constant weight before the dry weight of the plants and the dry weight of the roots.

### 2.4 Soil sample collection and processing

Upon the completion of the inquiry, namely on the 30 day, soil samples from the inter-root region were gathered from the OSF, OS, and CK experimental groups. The soil was carefully distributed in a controlled and sterile setting. The tomato seedlings were extracted using sterilized forceps, and the soil adhering to the tomato roots was delicately removed using a sterile soft-bristle brush, ensuring a distance of 1−2 mm from the root surface. Adequate amounts of soil located between the roots were collected into EP tubes. The dirt that remained was isolated from the roots and debris, transferred into a hermetically sealed container, and stored in a refrigeration unit at a temperature of roughly −20°C.

### 2.5 Soil physicochemical properties determination

In this phase of the investigation, the following experimental instruments were utilized: a portable pH meter (model PHS-3C), a portable conductivity meter (model 2265FS, manufactured in the United States), and a soil carbon and nitrogen analyzer (model Elementar Vario MAX CN, manufactured in Germany) for the determination of soil total nitrogen (TN) and total carbon (TC).

1. Soil water content (WC): a certain mass of fresh soil is weighed, desiccated to a constant weight at 105°C, and then the dry soil mass is weighed and calculated using the formula as follows:
  $$WC\left(\%\right)=\frac{M\,w-M\,t}{M\,w}\times 100$$
  In the equation, Mw is the mass of fresh soil (g); Mt is the mass of dried soil (g); and WC is the water content of the soil (%).
2. Soil pH and electrical conductivity (EC): A sample of soil was removed from the self-sealing bag, air-dried at room temperature, passed through a 2 mm sieve, mixed with deionized water at a ratio of 1:2.5, and stirred for 30 min. After 10 min, the soil pH and EC were measured directly using a portable pH meter (pHS-3C) and conductivity meter (2265FS, USA).
3. Determination of mineral nitrogen: 10.0 g of fresh soil samples were weighed and passed through a 2 mm sieve in a 50 mL centrifuge tube. Revise Su Tao’s literature (Su et al., 2005).
4. The carbon and nitrogen content of the soil was determined by adding a sample of air-dried soil that had been sieved through a 100 mesh sieve to a carbon and nitrogen analyzer. The analyzer was run following the provided instructions. Before determining the sample, it is necessary to make a subtraction of the blank value.

### 2.6 Analysis of rhizosphere soil bacterial community structure in tomato

#### 2.6.1 Extraction of genomic DNA and PCR amplification

The soil DNA was extracted in accordance with the methodology described by Kamdem et al. (2023). The genomic DNA was isolated from soil samples using the CTAB method, followed by an assessment of its purity and concentration using agarose gel electrophoresis. The DNA extract was diluted to a concentration of 1 nanogram per microliter (ng/μL) and employed as a template for polymerase chain reaction (PCR) amplification. The PCR reaction utilized Phusion^®^ High-Fidelity PCR Master Mix containing GC Buffer and high-performance, high-fidelity enzymes sourced from New England Biolabs. The study done by Zhang H. et al. (2019) examined the application of prokaryotic universal primers 341F and 806R. The primers and sequences utilized for PCR amplification were as described below:


$$\underline{\text{341F(CCTAYGGGRBGCASCAG)}}$$

$$\underline{\text{806R(GGACTACNNGGGTATCTAAT)}}$$

#### 2.6.2 PCR product purification and mixing

Libraries were constructed using Thermofisher’s Ion Plus Fragment Library Kit 48rxns library construction kit and then sequenced using Thermofisher’s Life Ion S5TM after completion of library construction, passing Qubit quantification, and library testing.

#### 2.6.3 Library construction and high-throughput sequencing

This study analyzed the bacterial community composition of tomato inter-rhizosphere soil using high-throughput sequencing. The V3 ∼ V4 regions in the 16S rDNA of the inter-root soil genome of tomatoes grown under CK, OS, and OSF treatments were sequenced and analyzed. In the reference study (Liu et al., 2022), Venn diagrams, PCA plots, Speraman correlation analysis heat maps, and CCA plots were developed based on high-throughput sequencing results to analyze the effects of oyster shell soil amendments on the community structure and diversity of rhizobacteria in acidic tomato soil.

### 2.7 Data processing and analysis

Using Excel 2016 and SPSS 24 statistical analysis software, the data were analyzed and assembled. To investigate the diversity of species composition in the various treatment groups, all effective tags were clustered into OTUs (operational taxonomic units) with 97% identity, and then the representative sequences of OTUs were annotated with species. The representative sequences for each OTU were then annotated. Spearman correlation was applied to examine the association between bacterial abundance and environmental factors.

## 3 Results and analysis

### 3.1 Effect of oyster shell soil conditioner on tomato cultivation

#### 3.1.1 Effect of oyster shell soil conditioner on tomato seed germination

The formulas in the experimental method were used to calculate the germination potential, germination rate, and germination index of tomato seeds. Different oyster shell soil conditioners had varying effects on the germination of tomato seeds, as shown in Table 1. The order of germination potential was OSF > OS > CK, with significant improvements of 24.99% in the OS group and 41.65% in the OSF group over the CK group; the order of germination rate was OSF = OS > CK, with significant improvements of 17.34% in both the OS and OSF groups over the CK group. The order of germination rate was OSF = OS > CK, and the germination rate of both OS and OSF groups was significantly increased by 17.34% relative to the CK group; the order of germination index was OS > OSF > CK, and it was significantly increased by 39.17% in the OS group and 36.86% in the OSF group compared to the CK group. In conclusion, the oyster shell soil conditioner supplemented with oyster shell powder was effective in promoting tomato seed germination in acidic red loam soil, and the OSF group was more effective in promoting germination.

**TABLE 1:** Effects of different treatment groups on tomato seed germination.

Sample	Germination potential/%	Germination rate/%	Germination index/%
CK	66.67 ± 3.33c	83.33 ± 3.33c	28.11 ± 3.53c
OS	83.33 ± 3.33b	97.78 ± 1.92a	39.12 ± 1.45a
OSF	94.44 ± 1.92a	97.78 ± 3.85a	38.47 ± 0.95b

a, b, and c indicate significant differences between treatment groups *p* < 0.05.

#### 3.1.2 Effect of oyster shell soil conditioner on the development of tomato seedlings

At the end of the 30th day of the experiment, plant height, root length, plant’s fresh weight, root’s fresh weight, plant’s dry weight, and root’s dry weight were measured for each treatment group; the results are shown in Table 2. The biomass data of the OS and OSF groups exhibited statistically significant improvements compared to the CK group, with a significant difference observed (*p* < 0.05). Moreover, there was no significant difference in biomass between the OSF and OS groups, suggesting that the primary constituent of calcium carbonate in both groups played a significant role in ameliorating soil acidity. Furthermore, the effectiveness of OS and OSF in improving soil conditions was found to be comparable.

**TABLE 2:** Effects of different treatment groups on tomato growth.

Sample	Plant height/cm	Root length/cm	Plant’s fresh weight/g	Root’s fresh weight/g	Plant’s dry weight/g	Root’s dry weight/g
CK	3.21 ± 0.45c	2.5 ± 0.57c	0.0297 ± 0.0065c	0.0034 ± 0.0013b	0.0022 ± 0.0007b	0.0008 ± 0.0005b
OS	5.68 ± 0.36b	3.15 ± 1.07b	0.1003 ± 0.0147a	0.0173 ± 0.0028a	0.0062 ± 0.0012a	0.004 ± 0.0015a
OSF	6.31 ± 0.67a	3.46 ± 1.03a	0.0985 ± 0.0101a	0.0185 ± 0.0039a	0.0062 ± 0.001a	0.0029 ± 0.0008a

a, b, and c indicate significant differences between treatment groups *p* < 0.05.

### 3.2 Effect of oyster shell soil conditioner application on the physicochemical properties of tomato inter-root soil

Oyster shell powder is made by grinding the shells of the marine organism oyster, which is alkaline in nature. Oyster shell powder mostly consists of calcium carbonate, a compound known for its excellent adsorption capabilities and ability to raise the soil’s pH level. Moreover, the rise in pH level creates the ideal physicochemical environment for acid-tolerant species to reproduce and metabolize soil organic components, resulting in an augmented biomass of microorganisms and soil respiration (Neale et al., 1997; Lee et al., 2008). Oyster shells are rich in minerals, proteoglycans, and various organic matter, such as sodium, barium, copper, iron, magnesium, manganese, nickel, and several trace elements. Incorporating oyster shell soil conditioner into the soil increases its content, thereby augmenting the availability of ionic minerals to plants. This enhancement facilitates plant growth and effectively improves the soil’s electrical conductivity. Additionally, it has been shown that the application of oyster shell soil conditioner leads to an increase in soil organic matter, rapid phosphorus availability, and exchangeable cation concentrations. Moreover, it has been found to dramatically enhance the levels of soil microbial carbon and nitrogen content, stimulate soil enzyme activity, and ultimately result in higher crop yields.

As shown in Table 3, the different treatment groups had different amounts of total nitrogen (TN), total carbon (TC), pH, electrical conductivity (EC), and water content (WC) in the soil. In comparison to the control group, the implementation of OS and OSF resulted in a substantial increase in soil total nitrogen (*p* < 0.05), soil total carbon (*p* < 0.05), soil pH (*p* < 0.05), and soil electrical conductivity (*p* < 0.05). In the two treatment groups, the percentage increase in soil total nitrogen was 16.2 and 59.9%, soil total carbon was 25.8 and 27.7%, pH was 56.9 and 55.9%, and conductivity was 377.5 and 311.5%, while soil water content varied. Compared to the control group, the OS group reduced the amount of The OS group experienced a 10.1% decrease in soil water content compared to the control group (*p* < 0.05), while the OSF group experienced fluctuations compared to the control group that were not significant changes (*p* > 0.05).

**TABLE 3:** Effects of different treatment groups on the physical and chemical properties of red soil.

Sample	TN/(g⋅kg^–1^)	TC/(g⋅kg^–1^)	pH	EC/(us⋅cm^–1^)	WC/(%)
CK	1.42 ± 0.11c	11.21 ± 0.55b	4.8 ± 0.02b	120 ± 25c	15.88 ± 1.06a
OS	1.65 ± 0.24b	14.1 ± 1.73a	7.53 ± 0.02a	573 ± 52a	14.27 ± 0.76b
OSF	2.27 ± 0.34a	14.31 ± 1.25a	7.48 ± 0.04a	494 ± 20b	15.74 ± 0.87a

a, b, c indicate significant differences between different treatment groups *p* < 0.05.

### 3.3 Effect of oyster shell soil conditioner application on the bacterial diversity of tomato inter-rhizosphere soil

In the field of microbial diversity research, investigators utilize two distinct spatial scales for analysis, namely alpha and beta. Alpha diversity examines the abundance and diversity of microorganisms within a given community through the application of single-sample diversity analysis. The aforementioned investigations encompass a variety of statistically assessed indices that facilitate the assessment of species abundance and diversity within ecological groups. Beta diversity is a measure of the variation in species composition between communities that occupy different habitats along an environmental gradient.

#### 3.3.1 The impact of oyster shell soil conditioner on the quantity and variety of bacteria in the inter-root soil of tomato plants

Table 4 displays the results of the alpha diversity analysis conducted on the bacterial diversity inside the soil next to the roots of tomato plants. The CK, OS, and OSF categories demonstrated a significant number of operational taxonomic units (OTUs), with counts of 1334, 1271, and 1153, respectively. The extent of library coverage for each of the three sample groups was found to be 99.5%. The extensive coverage seen in this study indicates a substantial likelihood of identifying gene sequences within the soil samples. This finding shows that the sequencing depth was adequate and that the resulting sequencing outcomes accurately represent the bacterial population present in the inter-rhizosphere soil of tomatoes.

**TABLE 4:** Bacterial diversity index of rhizosphere soil in different treatment groups.

Sample	Diversity analysis				
	**OTUs**	**Shannon**	**Chao1**	**ACE**	**Coverage**
CK	1334	7.761	1493.091	1503.759	99.5%
OS	1271	7.684	1384.282	1408.293	99.5%
OSF	1153	7.126	1293.671	1314.915	99.5%

Based on the findings presented in Table 4, it can be observed that the bacterial community’s diversity experienced a decrease in both the OS and OSF groups when compared to the control group. Specifically, the OS group exhibited a reduction of 4.7%, while the OSF group saw a more substantial decrease of 13.6%. Employing the Chao1 and ACE indices, the number of OUTs in the bacterial community and the diversity of the microbial community in the samples were estimated. Based on the Chao1 and ACE indices, the administration of either OS or OSF decreased the bacterial community’s abundance. OSF significantly reduced bacterial abundance compared to OS.

#### 3.3.2 Effect of oyster shell soil conditioner application on the OTU distribution of inter-root soil bacteria in tomatoes

**FIGURE 1:**
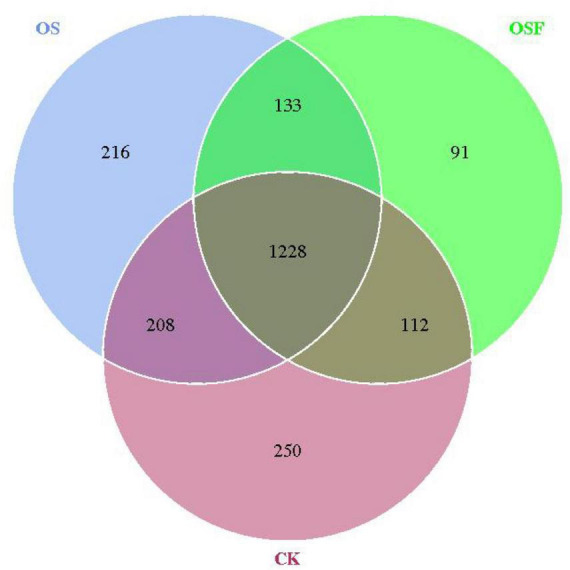
Venn of OTUs distribution of rhizosphere soil bacteria in different treatment groups.

The utilization of Venn diagrams proves to be a valuable method for evaluating the similarities and distinctions in the composition of operational taxonomic units (OTUs) between the treatment group employing oyster shell soil conditioner and the control group. Additionally, it allows for the analysis of the number of OTUs that are unique to individual samples and those that are shared among numerous samples. The root soil bacteria operational taxonomic unit (OTU) distribution chart for various treatment groups (Figure 1) demonstrates that the CK, OS, and OSF groups harbored 250, 216, and 91 distinct OTUs, respectively. Additionally, it is noteworthy that 1228 OTUs were found to be common among all three groups. The utilization of OS or OSF resulted in the introduction of new species and modifications in the community structure of bacteria residing in the inter-rhizosphere soil of tomato plants, with a more pronounced effect observed in the OS group.

#### 3.3.3 Effect of oyster shell soil conditioner application on bacterial community composition of tomato inter-root soil

##### 3.3.3.1 Soil bacterial colonies in the inter-root zone of tomato: composition analysis

**FIGURE 2:**
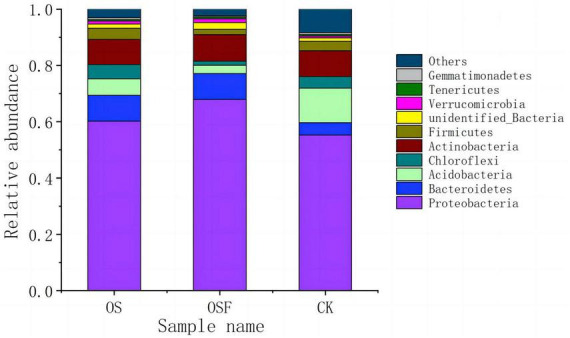
Community composition of bacterial phylum in rhizosphere soil of different treatment groups.

Figure 2 presents the makeup of soil bacterial communities at the phylum level, specifically focusing on the inter-root region. This figure illustrates the variations in population composition across different treatment settings. The relative abundance of the top 10 phyla in soil samples from the CK, OS, and OSF groups varied between 91.00 and 97.64%. The relative abundances of these 10 phyla were: *Proteobacteria* 55.32 ∼ 67.97%, *Bacteroidetes* 4.42∼9.22%, *Acidobacteria* 2.87 ∼ 12.18%, *Chloroflexi* 1.45 ∼ 5.12%, *Actinobacteria* 8.92 ∼ 9.46%, *Firmicutes* 1.98 ∼ 4.03%, *unidentified Bacteria* 1.27 ∼ 2.29%, *Verrucomicrobia* 0.67 ∼ 1.35%, and *Tenericutes* 0.45∼0.70%, and *Gemmatimonadetes* 0.39 ∼ 0.76%. *Proteobacteria*, *Bacteroidetes*, *Acidobacteria*, and *Actinobacteria* constituted more than 85% of the total number of samples and were the primary phylum in the three sample categories.

**FIGURE 3:**
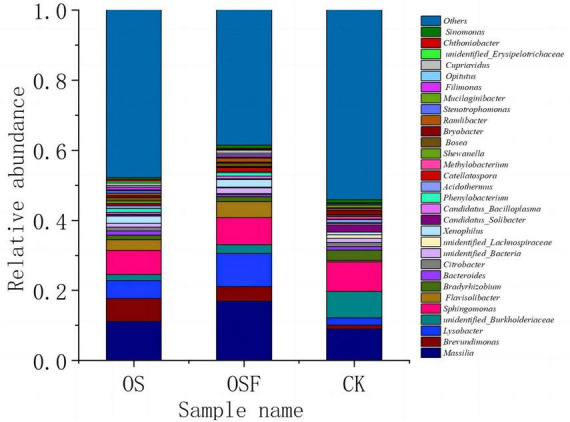
Community composition of bacterial genus in rhizosphere soil of different treatment groups.

Figure 3 illustrates the horizontal community composition of soil bacterial species across different treatment settings in the root soil. The relative abundance of the top 30 genera in each of the three categories ranged from 45.88 to 61.44% of the total genera. They are: *Massilia, Brevundimonas, Lysobacter, unidentified Burkholderiaceae, Sphingomonas, Flavisolibacter, Bradyrhizobium, Bacteroides, Citrobacter, unidentified_Bacteria, unidentified_Lachnospiraceae, Xenophilus, Candidatus_Solibacter, Candidatus_Bacilloplasma, phenylobacterium, Acidothermus, Catellatospora, Methylobacterium, Shewanella, Bosea, Bryobacter, Ramlibacter, Stenotrophomonas, Mucilaginibacter, Filimonas, Opitutus, Cupriavidus, unidentified_Erysipelotrichaceae, Chthoniobacter, Sinomonas*. Among them, *Massilia, Brevundimonas, Lysobacter, unidentified_Burkholderiaceae, Sphingomonas, Flavisolibacter, and Bradyrhizobium* were relatively abundant and were the dominant bacterial genera in tomato inter-rhizosphere soil.

##### 3.3.3.2 Effect of application of oyster shell soil amendment on the structure of bacterial genera in the inter-root soil of tomato

At the phylum level, *Proteobacteria*, *Bacteroidetes*, and *Acidobacteria* exhibited greater variability in the OS and OSF groups compared to the control group. Specifically, the relative abundance of *Proteobacteria* and *Bacteroidetes* increased, while the relative abundances of *Acidobacteria* and other phyla decreased. In the OS and OSF groups, the relative abundance of *Proteobacteria* increased from 55.3 to 60.2 and 68.0%, respectively. Moreover, the relative abundance of *Bacteroidetes* increased from 4.4 to 9.2%. Conversely, the relative abundance of *Acidobacteria* decreased from 12.2 to 5.8 and 2.9%, respectively. Additionally, the relative abundance of other phyla decreased from 8.3 to 9.2% in the OS and OSF groups.

At the genus level, the utilization of different oyster shell soil conditioners (OS and OSF) led to a significant alteration in the relative abundance of several bacterial genera compared to the control group. Specifically, there was a marked increase in the relative abundances of the genera *Massilia*, *Brevundimonas*, and *Lysobacter*, while a significant decrease was observed in the relative abundance of *unidentified_Burkholderiaceae*. The specific alterations were as follows: in the OS and OSF groups, the relative abundance of *Massilia* increased from 9.00 to 11.18 and 16.93%, respectively; the relative abundance of *Brevundimonas* increased from 1.13 to 6.56 and 4.14%, respectively; the relative abundance of *Lysobacter* increased from 2.00 to 5.12 and 9.49%; whereas the relative abundance of *unidentified_Burkholderiacea* decreased from 7.50 to 1.74 and 2.49%, respectively.

**FIGURE 4:**
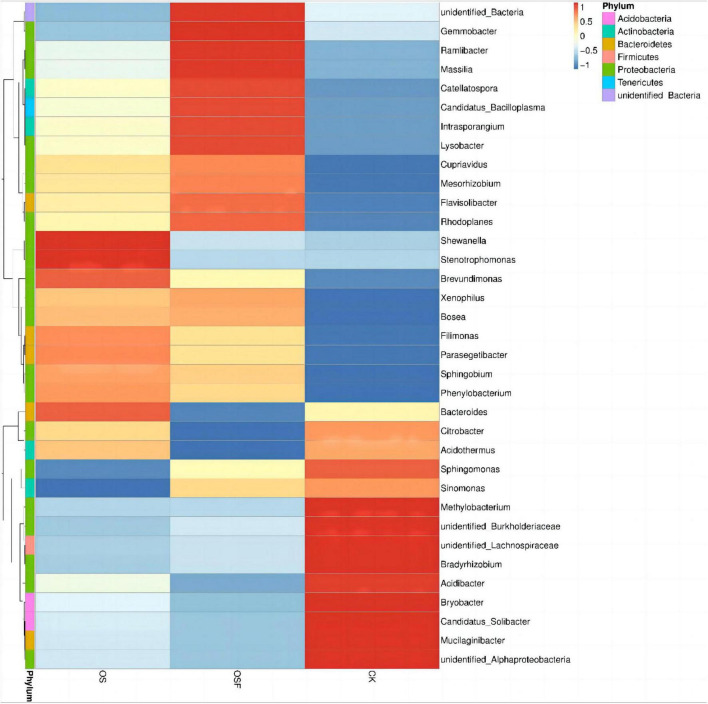
Cluster heat map of bacterial abundance in rhizosphere soil of different treatment groups.

In order to examine the variations in the predominant genera of soil bacteria in the inter-rhizosphere of tomatoes across different treatment groups, we selected the top 35 genera with significant relative abundance. This selection was based on the species annotation and abundance data of all samples at the genus level. The chosen genera were then clustered according to their abundance information and visualized as a heat map (Figure 4). This approach aimed to facilitate the identification of the extent of aggregation among different genera within the distinct treatment groups.

As shown in the heat map (Figure 4), there are differences at the genus level among the three dominant groups, CK, OS, and OSF. *Mucilaginibacter*, *Candidatus_Solibacter*, *Bryobacter, Acidibacter*, *Bradyrhizobium*, and *Methylobacterium* are the dominant genera in the CK group; *Shewanella* and *Stenotrophomonas* are the dominant genera in the OS group; and *Gemmobacter*, *Ramlibacter*, *Massilia*, *Catellatospora*, *Candidatus_Solibacte*r, *Intrasporangium*, and *Lysobacter* are the dominant genera in the OSF group. It can be observed that the relative abundance of various bacterial genera in distinct treatment groups varied slightly, as did their dominant genera.

The findings above suggest that the implementation of oyster shell soil conditioners, namely OS and OSF, had a considerable impact on the structure of the rhizosphere microbiome. This resulted in a modification of the microbial niche situated between plant roots, which varied in intensity and scope, thus indicating that the effects of OS and OSF on soil composition were distinguishable and independent of one another.

#### 3.3.4 Principal component effects of the application of oyster shell soil conditioner to the inter-root soil bacterial communities of tomato

**FIGURE 5:**
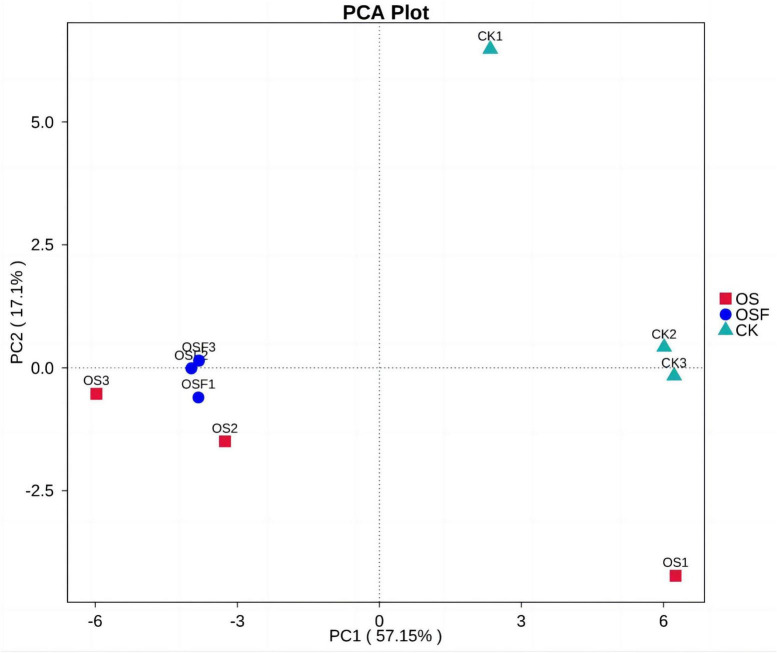
Principal component analysis (PCA) analysis of bacterial community composition in rhizosphere soil of different treatment groups.

In order to analyze the differences caused by oyster shell soil amendments on the soil bacterial community structure in the tomato rhizosphere, we performed principal component analysis (PCA) on the soil bacterial community composition of the different treatment groups using PCA analysis in beta diversity analysis; the results of the PCA analysis are shown in the PCA analysis graph (Figure 5).

The first two principal components (PC1 and PC2) were selected for the analysis of the three groups of tomato inter-root soils. The first principal component made up 57.15% of the difference between samples, and the second principal component made up 17.1% of the difference between samples. This means that these two principal components were the main reasons why the bacterial community structure of tomato inter-root soils was different. The graph depicts the communities of three parallel samples from each treatment group, with the dots representing different colors to indicate the variation in bacterial community structure. Notably, the degree of separation between the dots corresponds to the level of significance of the observed changes. The observed spatial separation between the OS and OSF groups, as measured along the principal component 1 axis (PC1), was found to be larger than that observed for the CK group. Similarly, the spatial separation between the OS and OSF groups along the principal component 2 axis (PC2) was also larger than that observed for the CK group. These findings suggest that both the OS and OSF groups have significantly influenced the composition and structure of bacterial communities.

### 3.4 Analysis of the correlation between inter-root soil bacterial species diversity and environmental factors in various treatment groups

#### 3.4.1 Spearman correlation analysis

**FIGURE 6:**
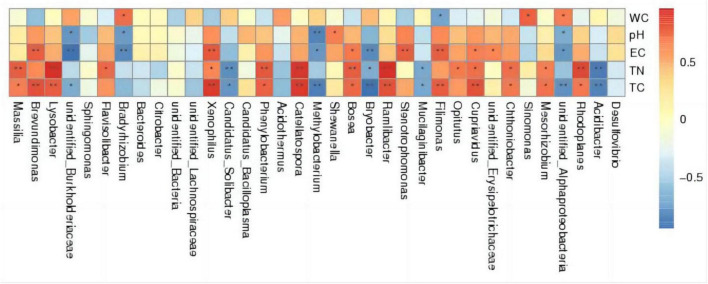
Spearman correlation analysis thermogram.

As depicted in the Spearman correlation analysis thermogram (Figure 6), the water content (WC) was positively correlated with *unidentified_Alphaproteobacteria*, *Sinomonas*, and *Bradyrhizobium* (*p* < 0.05) and negatively correlated with *Filimonas* (*p* < 0.05). pH had a significant positive correlation with Shewanella (*p* < 0.05); a negative correlation with *Bradyrhizobium*, *unidentified_Alphaproteobacteria*, and *unidentified_Burkholderiaceae* (*p* < 0.05); and a highly significant negative correlation with *Methylobacterium* (*p* < 0.01). Electroconductivity (EC) is highly positively correlated with *Filimonas*, *Stenotrophomonas*, etc. (*p* < 0.01), positively correlated with *Bosea*, etc. (*p* < 0.05), highly negatively correlated with *Bryobacter*, etc. (*p* < 0.01), and negatively correlated with *Methylobacterium*, etc. (*p* < 0.05). And for TC and TN, there are also some bacterial properties that are positively (or negatively) related in a very significant way. The aforementioned results indicate that total carbon (TC), total soil nitrogen (TN), electrical impact on thermal conductivity, soil pH, and water content have a great abundance of interracial soil bacteria, with varying effects on distinct bacteria.

#### 3.4.2 CCA analysis

**FIGURE 7:**
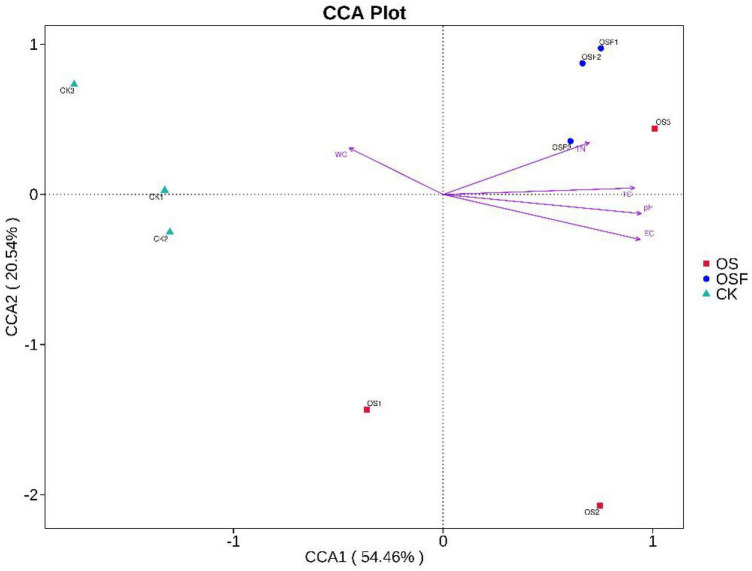
CCA analysis chart of different treatment group.

The distance between the points of the CK, OS, and OSF groups was farther in the CCA (Figure 7), indicating that the differences in bacterial community composition among the three groups were obvious. The angles of the control group and WC were acute and obtuse with pH, EC, TN, and TC, indicating that the control group was positively correlated with WC and negatively correlated with pH, EC, TN, and TC; the distribution of the OS group was more dispersed and all were obtuse with WC, indicating that the OS group was negatively correlated with WC, while the angles of the OS group were different with pH, EC, TN, and TC, with large differences; the OSF group was at an obtuse angle to WC and at an acute angle with pH, EC, TN, and TC, indicating that the OSF group was negatively correlated with WC and positively correlated with pH, EC, TN, and TC. Therefore, variations in the structural composition of the inter-rhizosphere soil bacterial community may be related to changes in soil physicochemical factors.

## 4 Discussion

### 4.1 Effect of oyster shell soil conditioner application on tomato

In the tomato cultivation experiment, seed germination and seedling growth were enhanced in the OS and OSF groups relative to the CK group. It is possible that the Ca^2+^ contained in the OS and OSF groups improved the acidic soil environment and stimulated the growth of tomato seedlings. Based on empirical findings, it has been observed that when a tomato plant experiences stress due to adverse conditions, there is a notable elevation in the concentration of Ca^2+^. This surge in Ca^2+^ concentration prompts the activation of calcium channels inside the plant, thereby exerting regulatory control over crucial physiological processes and augmenting the plant’s resilience to adverse circumstances (Zhang, 2019). This study examines the impact of utilizing oyster shell soil conditioner on the growth of tomato seedlings in acidic soil. It is hypothesized that one potential mechanism behind this observed growth promotion is the facilitation of Ca^2+^ channel initiation in tomato plants. However, Ca^2+^ is an important structural and functional part of plant chloroplasts and is involved in photosynthesis. Adding calcium from outside the plant increases its ability to synthesize food when it is exposed to abiotic stress (Zhang et al., 2014). Therefore, in acidic soils, the application of oyster shell soil conditioner promoted photosynthesis in tomatoes, which in turn promoted the growth of tomatoes. In addition, Ca^2+^ in soil can interact with P^5+^ and K^+^ ions to significantly improve the biological condition and tomato fruit quality during the growth of tomatoes (Xu, 2003). The synergistic effect of Ca^2+^ and other inorganic salt ions on tomato growth may be another reason why oyster shell soil conditioner promotes tomato growth. Furthermore, a variety of physiological ailments, including navel rot, fruit breaking, and poor coloring, may manifest during the growth of tomatoes in the absence of sufficient calcium (Li et al., 2019; Liu et al., 2019). However, the application of oyster shell soil conditioner can effectively ensure optimal growth conditions, facilitate successful blooming and fruiting, and enhance the overall quality of tomatoes. OS is solely calcium carbonate and contains a small amount of calcium oxide, which is too alkaline and may cause seedling burn if used improperly. OSF, on the other hand, is more suitable for very acidic soils, and the dosage must be calculated and rigorously controlled. When considering all factors, it can be concluded that the oyster shell soil conditioner developed by OSF demonstrated the highest level of effectiveness.

### 4.2 Effect of oyster shell soil conditioner application on bacterial diversity in acidic inter-root soil of tomato

The diversity of soil microorganisms has become an essential factor in maintaining soil function (Xu et al., 2010; Tan et al., 2014) and an important index for managing and evaluating soil quality (Fernandez et al., 2016; Li, 2018). According to studies, oyster shell powder can modify bacterial abundance and diversity, leading to a rise in bacterial abundance and diversity (Zhang, 2018). In this study, however, OS and OSF altered the relative abundance, diversity, and community structure of tomato soil bacteria, resulting in a slight reduction in bacterial community diversity and a different effect for each. It is hypothesized that this difference is due to the fact that the structure of the tomato inter-rooted bacterial community has not yet reached a stable state during the 30-day cultivation period, and studies have shown that this structure stabilized gradually between 60 and 70 days (Cheng, 2018). It is also possible that OS and OSF altered the soil pH too drastically for too short a period of time, resulting in the mass extinction of the original acid-tolerant bacterial population in the inter-rooted soil, whereas the bacterial community that adapted to the new environment did not recover within a short period of time. Additionally, bacterial diversity and relative abundance were greater in the OSF group than in the OS group, most likely due to the presence of organic microbial fertilizer in the OSF group, which is consistent with previous research (Xiang et al., 2021).

### 4.3 Effect of oyster shell soil conditioner application on bacterial community structure of tomato acidic inter-root soil

The introduction of oyster shell soil conditioner into acidic soils has the potential to modify the structure of the soil microbial community and facilitate its evolutionary processes (Ji, 2020). Cheng (2018) employed high-throughput sequencing techniques to identify the predominant microbial communities present in the inter-rhizosphere soils of tomatoes. The results revealed that *Acidobacteria*, *Bacteroidetes*, *Gemmatimonadetes*, and *Proteobacteria* were the primary microbial taxa observed. *Proteobacteria* constituted the predominant species in the inter-rhizosphere soil of tomatoes, comprising approximately 34.15% of the bacterial population in this particular soil environment. Previous research has demonstrated the substantial contribution of *Proteobacteria* to soil remediation and the enhancement of nitrogen fertilizer utilization (Zhang et al., 2012; Xu et al., 2017). The phylum identified in this study included the above phylum after the application of oyster shell soil conditioner powder, and the relative abundance of *Proteobacteria* was the highest, consistent with previous discoveries. In addition, the relative abundance of *Bacteroidetes* increased significantly in the OS and OSF groups compared to the CK group, indicating that the relative abundance of *Bacteroidetes* was greater in healthy tomatoes than in diseased tomatoes (Shen et al., 2021), indicating that the application of oyster shell soil conditioner increased the number of dominant *Bacteroidetes* in tomato inter-root soil and promoted tomato growth. The impact of OSF was marginally more significant compared to OS, presumably due to the inclusion of organic microbial fertilizer in OSF. This component has the ability to assimilate a diverse range of advantageous bacteria, nutrients, and trace elements that facilitate the development of tomatoes. Consequently, this fosters the proliferation and reproductive capabilities of rhizosphere bacteria (Rousk et al., 2010; Wu et al., 2019).

At the taxonomic level of genus, the present study observed that the application of oyster shell soil conditioners OS and OSF resulted in an increase in the relative abundance of *Massilia* from 9 to 11.18 and 16.93%, respectively. Similarly, the relative abundance of *Brevundimonas* grew from 1.13 to 6.56 and 4.14% with the application of OS and OSF, respectively. Additionally, the relative abundance of *Lysobacter* increased from 2 to 5.12 and 9.49% with the application of OS and OSF, respectively.

The principal constituent of oyster shell powder, according to our previous analysis, is calcium carbonate, which alters the soil’s pH when applied to the soil. According to previous studies, changes in soil pH have a cascading effect, i.e., fluctuations in soil pH may trigger changes in a range of soil nutrients, including, but not limited to, microbial-dominated soil nitrogen turnover, soil phosphorus solubility, soil fast-acting potassium content, and soil organic matter. In addition, the pH fluctuations also affect the microbial indicators in the soil (Wang et al., 2013; Reza et al., 2016; Penn and Camberato, 2019; Wu et al., 2023). Therefore, we suppose that the change in soil pH after the application of oyster shell soil conditioner was the main reason for the change in the occupancy structure of bacterial genera in the soil.

At the phylum level, the community structure of tomato inter-rhizosphere soil didn’t change much. However, the genus distribution heat map of tomato inter-rhizosphere soil showed that the genus varied a lot between the applied treatments, and each sample treatment had its own dominant genus. Although the principal components of OS and OSF are identical, the principal dominant genera of the two are distinctive. It’s possible that OSF absorbs related probiotics that have antagonistic or synergistic effects on inter-rhizosphere bacterial populations. It’s also possible that other nutrients or trace elements that OSF absorbs have some effect on inter-rhizosphere bacterial growth that helps it grow. Finally, OSF may cause tomato roots to make more secretions that affect inter-rhizosphere bacterial growth (Zhalnina et al., 2015). When oyster shell soil conditioner was put on tomato inter-rhizosphere soil, it changed the soil’s physical and chemical properties and acted on microorganisms. This caused changes in the species and relative abundance of dominant genera in the soil, which in turn changed the structure and distribution of soil bacterial communities.

### 4.4 Correlation between the application of oyster shell soil conditioner on microorganisms and environmental factors in acidic tomato inter-root soil

The soil’s microbial activity, abundance, and community structure interact with plant growth and a variety of parameters like carbon and nitrogen content, pH, and electrical conductivity. The number, species, and composition of inter-rooted microorganisms can vary according to environmental factors (Andrew et al., 2012). The principal soil parameter that determines the diversity and biomass of soil microorganisms is pH, which influences the microbial community by modulating the efficacy of soil nutrients (Zhalnina et al., 2015). In this experiment, pH in the experimental group was positively correlated with *Shewanella* (*p* < 0.05) and negatively correlated with *Bradyrhizobium*, *unidentified_Alphaproteobacteria*, and *unidentified_Burkholderiaceae* (*p* < 0.01), showing a highly significant negative correlation with *Methylobacterium* (*p* < 0.01). All of the aforementioned genera belong to the *Proteobacteria*, and it is assumed that the bacteria of the *Proteobacteria* may be closely related to the regulation of soil pH. This is consistent with previous studies (Cheng, 2018).

### 4.5 Efficacy of oyster shell soil conditioner application for plant disease control

Previous studies (Ofek et al., 2012; Naqqash et al., 2020; Lin et al., 2021) have demonstrated that *Massilia*, *Lysobacter*, and *Brevundimonas* exhibit beneficial effects on plants, including the induction of resistance mechanisms and promotion of growth. These bacterial species can be classified as helpful bacteria. The *Burkholderiaceae*, conversely, represent a clearly delineated genus of pathogenic bacteria. Within this genus, many phytopathogenic bacteria are known to induce distinct manifestations of plant diseases, such as rot, wilt, and extensive necrosis.

In this study, the relative abundance of *Massilia*, *Brevundimonas*, and *Lysobacter* increased significantly after the application of oyster shell soil conditioner, while the relative abundance of *unidentified_Burkholderiaceae* decreased most significantly. As mentioned in the previous paper (Carrión et al., 2018; Gu et al., 2019), the addition of oyster shell soil conditioner to acidic soil will change the community structure of soil microorganisms, and the change in community structure is also important for the control of plant disease. According to previous studies, *Massilia* plays an important role in soil remediation and amelioration. Feng et al. (2016) found a new *Massilia* that can produce dimethyl disulfide (DMDS), which can be used as an alternative fumigant to control the high infection levels of soilborne pathogens, nematodes, and weeds. DMDS can also degrade PAH phenanthrene and chloroacetamide-based herbicides, produce heavy metal resistance, and have phosphorus solubilization (Islam et al., 2019; Yang et al., 2019) showed that isolates of *Brevundimonas* can promote the growth of tomato plants, act as an effective biocontrol agent against tomato wilt, and play an important role in soil toxicity removal (Gu et al., 2019). *Lysobacter* is not only able to colonize the inter-root zone of many plants but also secretes a variety of antibiotics, extracellular hydrolytic enzymes, and biosurfactants to inhibit the growth of pathogens, thus controlling plant diseases (Ji, 2011), and *Lysobacter* strains are able to produce a type of antibiotic that has a pronounced antagonistic effect on seedling blight (Hashidoko et al., 1999; Yu et al., 2007). Kobayashi and Yuen (2005) studied enzyme-producing lysogenic bacilli and found a new antibiotic substance with heat stability that altered the morphology of the mycelium by regulating the synthesis pathway of ceramidase to control the disease. In this study, *unidentified_Burkholderiaceae* showed the most significant decrease in relative abundance. Burkholderia is an important group of plant pathogenic bacteria, including eight different species of phytopathogenic bacteria, and varies according to the specific host plant as well as to a particular occasion and cultivar (Zhang, 2016). Individual species have also been found to be closely related to plant roots (Urakami et al., 1994). After the introduction of oyster shell soil conditioner, we observed significant changes in the relative abundance of the four plant genera mentioned above. Based on examples from previous studies (Shen et al., 2018), microbial abundance and diversity were highly significant and negatively correlated with the incidence of siderophore diseases. Consequently, we deduce that the application of oyster shell soil conditioner led to a steady increase in the presence of beneficial genera, resulting in the suppression of detrimental genera. This was the underlying cause for the observed drop in the overall relative abundance. These data suggest that using oyster shell soil conditioner can improve the ability of tomato plants to fight diseases.

### 4.6 Main data analysis methods used in the article

The present study utilized various statistical techniques, such as the Shannon index, Spearman correlation, CCA chart, and PAC chart, to examine and interpret the experimental data. The Shannon index was utilized to estimate the diversity of the microbial population in the samples, where higher values corresponded to increased levels of diversity. In the figure depicting the Principal Component Analysis (PCA) analysis (Figure 5), The distance observed on the principal component axis 1 (PC1) between the OS group and the OSF group was significantly greater than that of the CK group. Similarly, the spatial distance between the two groups on the principal component axis 2 (PC2) was also significantly greater than that of the CK group. These findings suggest that both the OS group and the OSF group have altered the composition and structure of the bacterial community. However, the spatial distribution difference between the OS group and the OSF group on the two axes was relatively small, indicating a limited dissimilarity in community composition between these two groups. The disparity in structure is minimal. The observed phenomenon can be attributed to the predominant constituents of OS and OSF, namely calcium carbonate. These constituents primarily influence the structure and diversity of the bacterial population by modulating the pH levels of the soil. Spearman’s correlation analysis was used to figure out how environmental factors, microbial species, and the number of them affected each other. The *p*-values typically showed the correlation and significance between the two. CCA plots can be used to detect relationships between environmental factors, between samples and colonies, or between two, and to identify the most influential environmental factors on the distribution of samples.

## 5 Conclusion

The utilization of oyster shell soil conditioner facilitated the germination and development of tomato seedlings, with oyster shell flour (OSF) demonstrating greater overall efficacy compared to oyster shell (OS).

The Shannon index showed that applying OS and OSF to soil decreased the variety of microbes compared to the control group. The OSF group had the biggest drop, at 13.6%.

Between 91.00 and 97.64% of the dirt around tomato plants belonged to the ten most common phyla. A total of 85% of the main groups in the inter-rhizosphere soil of tomatoes were *Proteobacteria*, *Bacteroidetes*, *Acidobacteria*, and *Actinobacteria*.

When oyster shell soil conditioner was added, the relative abundance of three helpful genera—*Massilia*, *Brevundimonas*, and *Lysobacter*—increased to varying degrees. On the other hand, the relative abundance of the disease-causing *Burkholderiaceae* dropped by a large amount.

Environmental factors were correlated with the relative abundance of soil bacteria in the inter-root zone of tomatoes. pH was positively correlated with *Shewanella* (*p* < 0.05), negatively correlated with *Bradyrhizobium*, *unidentified Alphaproteobacteria*, and *unidentified_Burkholderiaceae* (*p* < 0.05), and highly significantly negatively correlated with *Methylobacterium* (*p* < 0.01).

## Data availability statement

The datasets presented in this study can be found in online repositories. The names of the repository/repositories and accession number(s) can be found in the article/supplementary material.

## Author contributions

YZ: Writing – original draft, Writing – review & editing. CY: Writing – review & editing, Writing – original draft. YX: Writing – review & editing. TY: Writing – review & editing. SW: Writing – review & editing.
